# A pathogenic *NR2F1* gene variant disrupts transcriptional activity and causes severe neurodevelopmental delay in Bosch-Boonstra-Schaaf syndrome

**DOI:** 10.1186/s41065-025-00394-8

**Published:** 2025-03-01

**Authors:** Juan Liu, Jihong Hu, Pingqiu Zhou, Yaqin Duan, Shuigui Yin

**Affiliations:** Department of Rehabilitation, The Affiliated Children’s Hospital of Xiangya School of Medicine, Central South University (Hunan children’s hospital), Ziyuan Road & No. 86, Changsha, 410001 Hunan China

**Keywords:** NR2F1 variant, BBSOAS, Neurodevelopmental disorders, Optic atrophy

## Abstract

**Introduction:**

Nuclear receptor subfamily 2, group F, member 1 (*NR2F1*) gene variations are associated with Bosch–Boonstra–Schaaf optic atrophy syndrome. The NR2F1 genotype correlates with its phenotype; variants within the DNA-binding domain may cause severe psychomotor developmental disorders. However, the mechanisms underlying these phenotypes remain unclear.

**Methods:**

Whole-exome sequencing was performed on the proband and her parents DNA. Candidate variants were verified by Sanger sequencing and bioinformatics analyses. Molecular dynamics simulations were performed to predict structural changes in the mutant NR2F1 protein. A dual-luciferase assay was used to analyze the variant’s effect on transcriptional activation.

**Results:**

The proband was a 10-month-old girl with severe motor and cognitive developmental delays accompanied by bilateral optic nerve pallor. Genetic testing revealed a novel *NR2F1* gene variant, NM_005654.6: c.452T > A (p.Met151Lys). Bioinformatics analysis suggested that this variant alters the protein structure or function. The molecular dynamics analysis showed that this variant might affect the stability of the zinc finger structure within the NR2F1 DNA-binding domain. Dual-luciferase assays indicated this variant affects transcriptional activation.

**Conclusions:**

The *NR2F1* variant c.452T > A (p.Met151Lys) may genetically cause the severe clinical phenotypes observed in this patient. This finding expands the spectrum of *NR2F1* variants.

**Supplementary Information:**

The online version contains supplementary material available at 10.1186/s41065-025-00394-8.

## Introduction

Neurodevelopmental disorders (NDDs) are highly heterogeneous diseases with complex etiologies involving genetic, environmental, immune, and metabolic factors, among which genetic factors dominate [1]. Bosch–Boonstra–Schaaf optic atrophy syndrome (BBSOAS, OMIM#615722) is an autosomal dominant inherited NDD first reported in 2014 [2]. BBSOAS exhibits diverse clinical manifestations characterized by various degrees of intellectual disability, developmental delay, and visual impairment. Hypotonia, seizures, speech difficulties, and autism spectrum disorder are also common [3]. The global incidence of BBSOAS is estimated to be about 1/100,000–1/200,000; however, approximately 100 patients have been diagnosed with BBSOAS [4]. Few Chinese patients have been reported [5–7]. Variants in the nuclear receptor subfamily 2, group F, member 1 gene (*NR2F1*) can cause BBSOAS. *NR2F1* encodes a transcription regulator that acts as a dimer to activate or inhibit target gene expression [8]. Although many patients with BBSOAS exhibit similar phenotypic characteristics, the severity varies; it may depend on the type and location of the *NR2F1* gene variants, suggesting the phenotype and genotype correlate in this disease [3].

This study performed a genetic analysis of a Chinese family with NDD. The proband presented with extremely severe psychomotor developmental delay in early childhood. Genetic testing revealed a de novo variant in the *NR2F1* gene in the protein’s DNA-binding domain (DBD). In vitro functional experiments have shown that this change may impair gene activation and transcriptional activity. Through this family study, we aimed to expand the *NR2F1* gene variant and phenotype spectrum in the Chinese population and raise clinical awareness of BBSOAS.

## Materials and methods

### Ethical statement and clinical evaluation

This family was referred to our department between 2022 and 2023. We collected general clinical information on the proband and obtained informed consent from her legal guardian to use her clinical data, imaging, and genetic information. The Medical Ethics Committee of Hunan Children’s Hospital approved the study (HCHLL-2023-100).

### Whole exome sequencing

Peripheral venous blood (3 mL) was collected from the patient and her parents into EDTA-K2 anticoagulant tubes. Genomic DNA was extracted using a DNA extraction kit (TIANGEN Biotech; Beijing, China), following the manufacturer’s instructions. The DNA was fragmented and used to construct a whole-exome library. The library was then hybridized with xGen Exome Research Panel v2.0 capture probes (Integrated Device Technology; IA, USA) for target enrichment. Following enrichment, the targeted DNA fragments were sequenced using the NovaSeq 6000 sequencing platform (Illumina) to identify possible gene variants.

### Bioinformatics analysis

The sequencing data were aligned to the human genome reference sequence version 19 using the BWA v0.7.17 software. Candidate gene variants were selected based on their frequency in databases, including the dbSNP, ExAC, and 1000 Genomes databases. GATK v.3.3.0 software was used to identify single-nucleotide variants and insertions/deletions. Online tools (SIFT, Polyphen2, MutationTaster, and M-CAP) were used to predict a variant’s effect on protein function. The presence of variants in the PubMed, HGMD, and ClinVar databases was also investigated. The American College of Medical Genetics and Genomics (ACMG) guidelines were used to assess the pathogenicity of the variants [9].

### Sanger sequencing

Primers targeting the *NR2F1* gene were designed based on sequences in the Ensemble database. The forward primer sequence was 5′-GCTTCCTGACCTGGTACAGG-3′; the reverse primer was 5′-CAGGACATCCAGGTGCTCTT-3′. The DNA was analyzed by Sanger sequencing using an ABI 3500XL sequencer (Applied Biosystems).

### Molecular dynamics simulation

The NR2F1 protein structure was compiled using the AlphaFold database (https://alphafold.ebi.ac.uk/). Molecular dynamics (MD) simulations were performed using GROMACS 5.14 with the Gromos53a6 force field. Simulations of 50 ns were run in a simple point charge water model, with 50 ps position restraints applied to the wild-type (WT) and mutant (MUT) structures. The root-mean-square deviation (RMSD) between the WT and MUT protein structures was calculated using the gmx RMS command. Structural differences were visualized using PyMOL v2.5.

### Cell culture

HEK293T cells were cultured in Dulbecco’s modified Eagle’s medium supplemented with 10% fetal bovine serum and 1% penicillin/streptomycin. The cells were incubated at 37 °C in a 5% CO_2_ incubator. Cells were used when they reached approximately 60–70% confluency.

### Plasmids construction

The coding region of the human *NR2F1* gene (NM_005654.6) was cloned into the pECMV-3×FLAG-N vector containing an N-terminal FLAG tag. The primer sequences are provided in Supplementary Material 1. The gene variant was introduced into the WT expression plasmid using a Q5 site-directed mutagenesis kit (New England Biolabs), according to the manufacturer’s instructions. The promoter sequence of EGR1 (a target of NR2F1) was cloned into the firefly luciferase–containing pGL3-basic vector to generate pGL3-basic-EGR1. This construct was co-transfected into HEK293T cells with pNR2F1-WT, pNR2F1-MUT, or pNR2F1-NC. The Renilla luciferase–containing pRL-TK plasmid served as an internal control. The transfection reagent was Lipofectamine 3000 (Thermo Fisher Scientific).

### Dual-luciferase assay

Firefly and Renilla luciferase activities were measured using the Dual-Luciferase Reporter Assay System (#E1910, Promega; Madison, WI, USA) according to the manufacturer’s instructions. Each experiment was performed in triplicate.

### Statistical analysis

Data are presented as means ± standard deviations. One-way analysis of variance was used for the statistical analysis. All statistical analyses and graphs were generated using GraphPad Prism software (version 8.0). Statistical significance was set at *P* < 0.05.

## Results

### Clinical phenotype

**Fig. 1:**
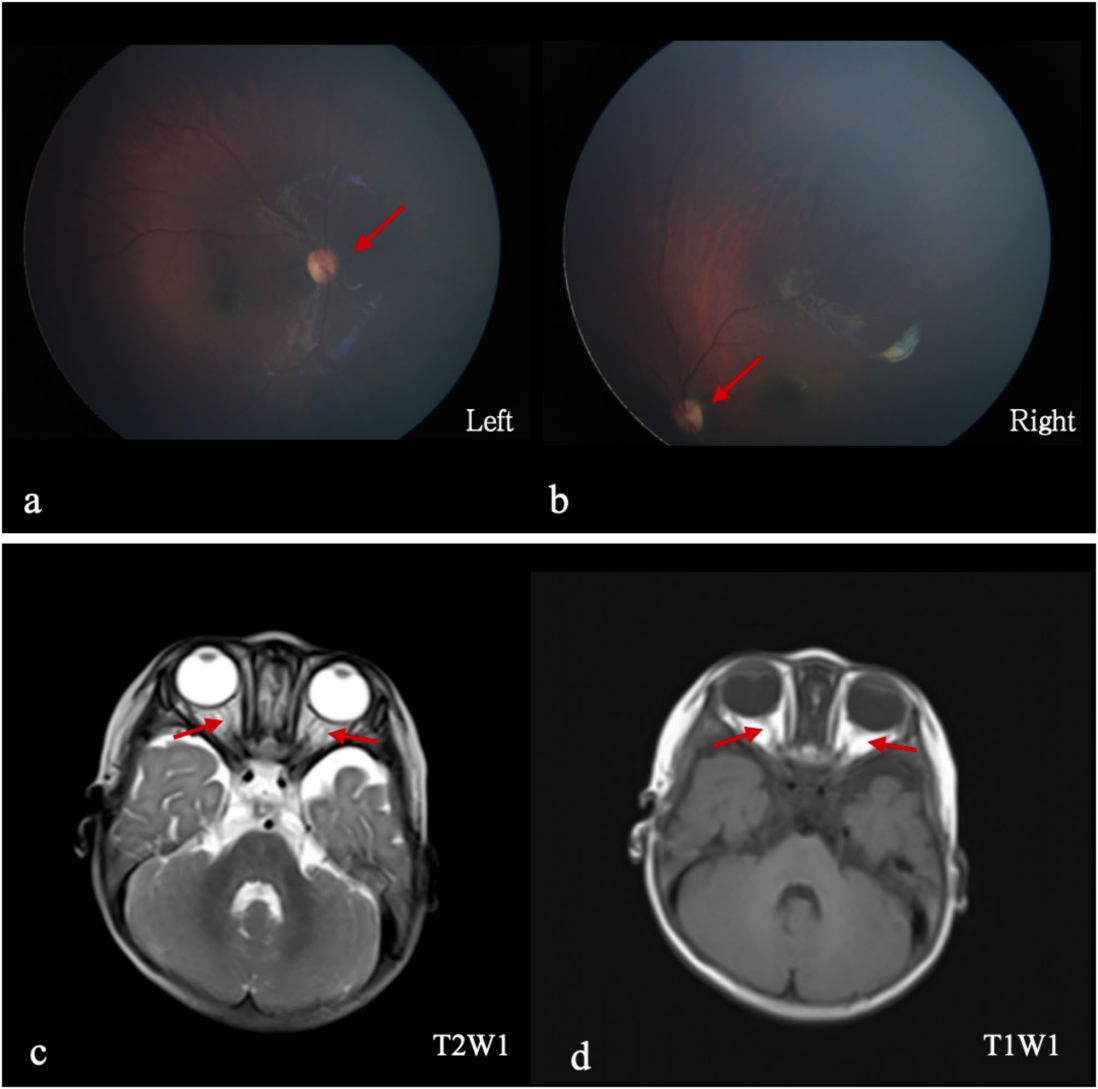
Imaging examinations. Fundoscopic examination of the patient shows bilateral optic nerve pallor (**a**,** b**). No significant optic nerve atrophy is observed on magnetic resonance imaging scans (**c**,** d**)

The patient was a 10-month-old female infant, her mother’s first child, born via vaginal delivery at 40 + 5 weeks of gestation. Her birth weight was 2,750 g, with an Apgar score of 10. She was admitted to the hospital for 8 days because of an unknown “neonatal infection and aspiration pneumonia” after birth because of meconium-stained amniotic fluid. The patient underwent rehabilitation training at another hospital for developmental delay at 5 months of age but showed a poor response. At 10 months of age, she could not sit independently, crawl, or hold her head steadily. Her ability to follow sounds and sight was poor, and she could not grasp objects voluntarily. She was thin on physical examination (height 73.2 cm, Z-score − 2.32; weight 7.28 kg, Z-score − 1.30). She occasionally gazed briefly at people or objects; making her laugh was difficult. She showed normal skin color with no abnormal facial features. The spine and limbs showed no malformations. She had increased muscle tone in both upper limbs and decreased muscle tone in both lower limbs. The Gesell and Peabody Developmental Motor Scales indicated severe developmental delays (Table 1). Imaging examinations and echocardiography revealed a patent foramen ovale; brain magnetic resonance imaging showed no obvious abnormalities. Fundoscopic examination of both eyes revealed bilateral optic nerve pallor (Fig. 1). The parents were healthy and unrelated. The patient had no family history of genetic disorders.

**Table 1:** Developmental assessment of the patient

Project	Developmental assessment
Gesell Child Development Scale	
Adaptability	DA: 4 weeks; DQ: 9 points; severe developmental retardation suggested
Great movement	DA: 12 weeks; DQ: 22 points; severe developmental retardation suggested
Fine motor	DA: 8 weeks; DQ: 21 points; severe developmental retardation suggested
Personal socialization	Not evaluated
Language ability	Not evaluated
Peabody Motor Development Scale	
Reflex	About 2-month-old
Posture	About 2-month-old
Move	About 2-month-old
Grip	About 2-month-old
Vision– Motion	Not evaluated
GMQ	51 points, with a percentile < 1%
FMQ	49 points, with a percentile < 1%
TMQ	45 points, with a percentile < 1%

DA: developmental age, DQ: developmental quotient, GMQ: gross motor quotient, FMQ: fine motor quotient, TMQ: total motor quotient

### Genetic testing results

**Fig. 2:**
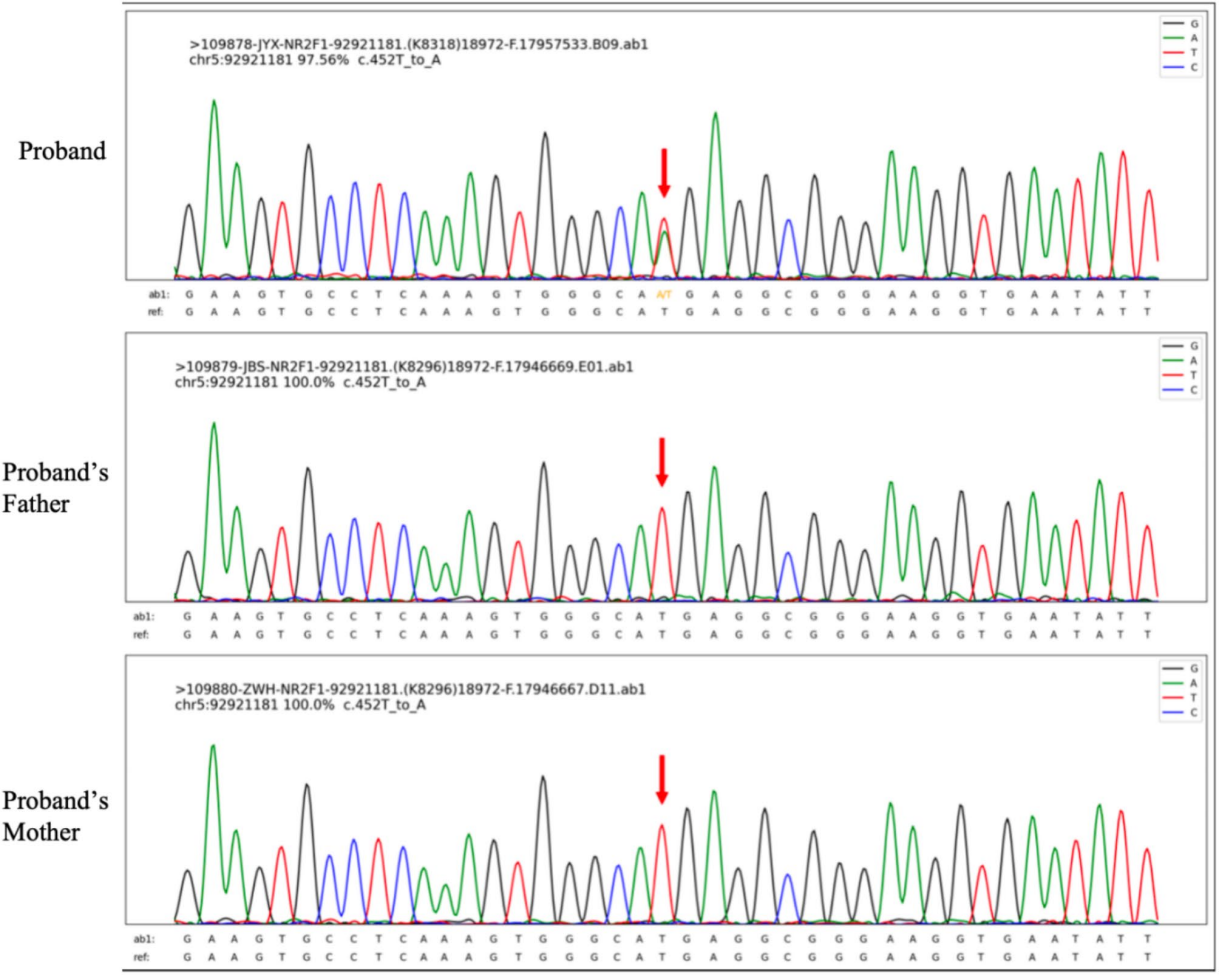
DNA sequencing. Sanger sequencing results show that the proband’s *NR2F1* gene has a de novo variant, NM_005654.6: c.452T > A (p.Met151Lys); both parents are wild type

**Fig. 3:**
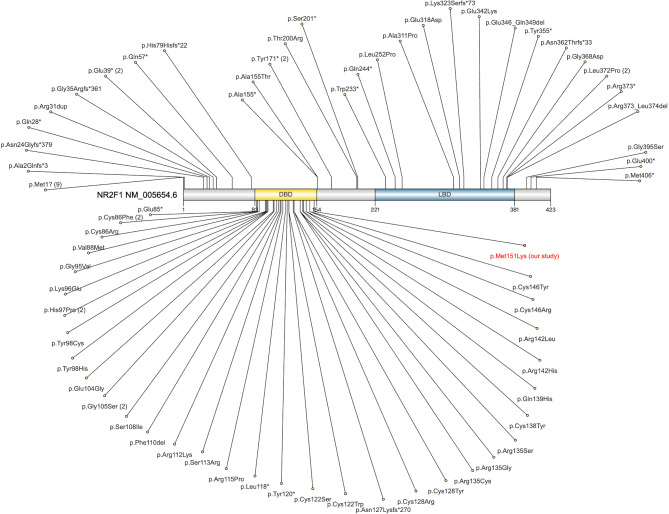
Spectrum of *NR2F1* gene variants reported in the literatures (red: present variant). The number in parentheses indicates the number of times the variant has been reported

The average sequencing depth in this study was 194 ×, with 99.7% of the target regions having a coverage depth greater than 20 ×. The logic for variant filtering is shown in Supplementary Material 2. Whole-exome sequencing identified a PS2 missense variant in the patient’s *NR2F1* gene (NM_005654.6: c.452T > A; p.Met151Lys). Both parents were wild type, suggesting a de novo variant. This variant was confirmed by Sanger sequencing (Fig. 2). The dbSNP, ExAC, and 1000 Genomes databases did not include this variant, indicating that it is a low-frequency variant (PM2). The variant has been included in the ClinVar database (Accession: VCV000988711.1) and is located in an exon region of the *NR2F1* gene where no benign variants have been reported (PM1). A search of the PubMed database revealed no clinical reports of this variant (Fig. 3 and Supplementary Material 3). Among predictive algorithms, the SIFT score was 0.001 (damaging), the Polyphen2_HDIV score was 0.043 (benign), the Polyphen2_HVAR score was 0.044 (benign), the MutationTaster score was 1 (disease-causing), and the M-CAP score was 0.939239 (damaging) (PP3). According to ACMG standards and guidelines, this variant is likely pathogenic.

### Effect of p.Met151Lys on transcriptional activity

**Fig. 4:**
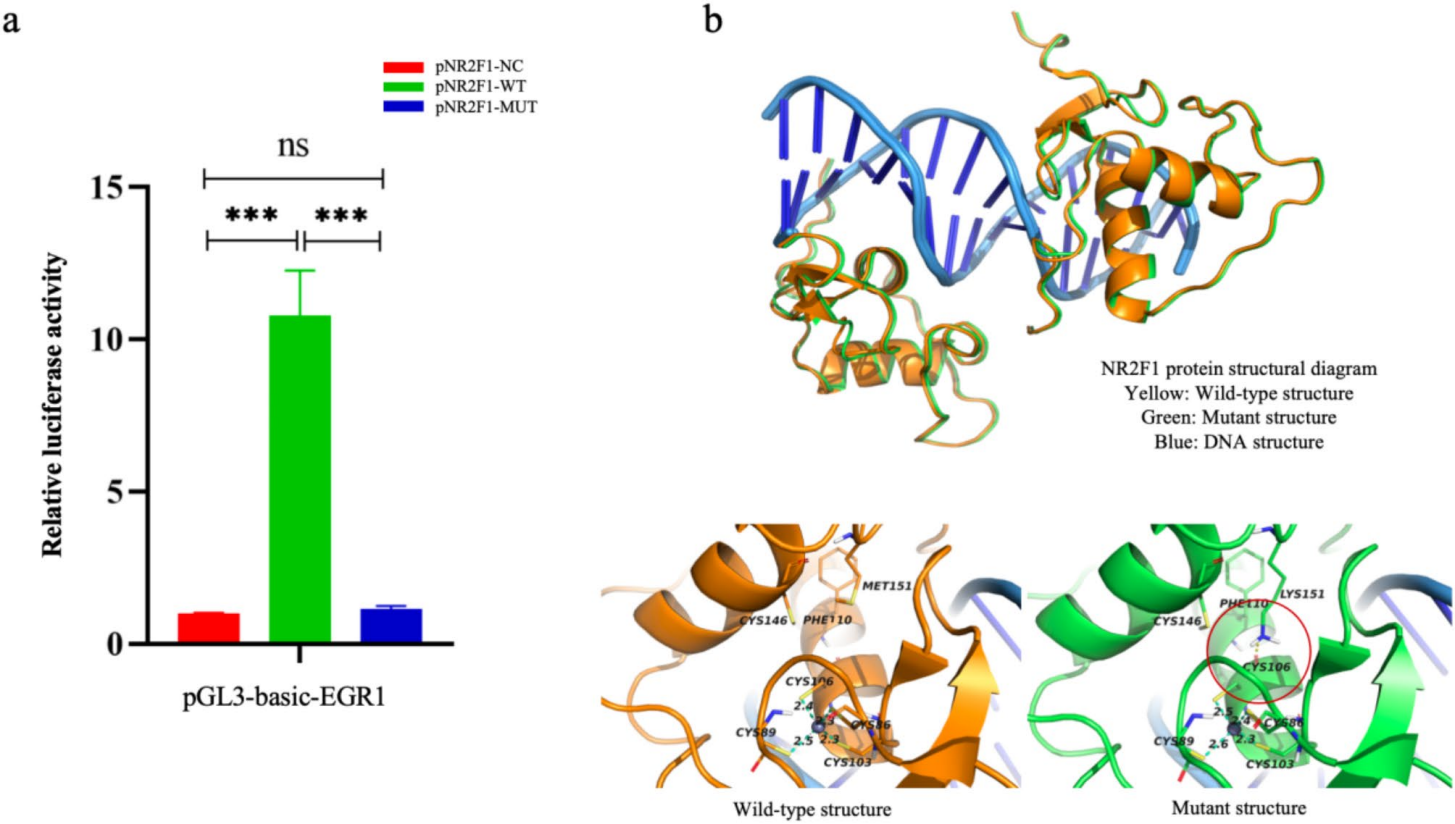
Variation analysis. **(a)** In vitro dual-luciferase assays show the *NR2F1* variant lowers transcriptional activation of EGR1, *** *P* < 0.001; **(b)** Molecular dynamics shows the changes in NR2F1 protein with and without the variant; the red circle indicates a local structural change

The dual-luciferase assay showed a significant increase in the fluorescence intensity from pNR2F1-WT compared to the empty vector control pNR2F1-NC. The fluorescence intensity of the variant pNR2F1-MUT did not differ significantly from that of pNR2F1-NC. These data suggest that the variant affects the transcription of EGR1 (Fig. 4a).

### MD suggests p.Met151Lys affects the zinc finger structures

The RMSD metric assesses whether a system has reached equilibrium and evaluates the average deviation between WT and variant structures [10]. The MD results showed that the WT and variant structures reached equilibrium at an RMSD value of 1.50 Å. Structurally, WT NR2F1 contains two highly conserved zinc fingers (ZF) that bind the DNA double helix. Each ZF domain contains four Cys residues that coordinate with a zinc ion [11]. Met151 is located in a loop structure. In the p.Met151Lys variant, the Lys side chain formed a hydrogen bond with Cys106 in ZF1 (Cys86-Cys89-Cys103-Cys106), increasing the zinc ion coordination distance. This finding suggests that this variant affects the structural stability of ZF1 (Fig. 4b).

## Discussion

This study focused on a young infant presenting with extremely severe psychomotor developmental delay and bilateral optic nerve pallor, indicating optic atrophy. These features suggested a diagnosis of BBSOAS. Genetic testing detected the presence of a de novo variant in the infant’s *NR2F1* gene. Dual-luciferase reporter assays showed this variant alters the transcriptional activation of EGR1. Although BBSOAS has been widely reported, additional cases with severe phenotypes strengthen the evidence that the genotype and phenotype correlate, facilitating further exploration of BBSOAS etiology and the identification of novel drug targets [4–7].

The NR2F1 protein was first identified as regulating chicken ovalbumin gene expression by binding directly to its promoter region; thus, it is also known as the chicken ovalbumin upstream promoter transcription factor [12]. Bosch et al. reported that *NR2F1* gene defects can cause NDD syndrome with optic atrophy, known as BBSOAS. BBSOAS has been widely reported globally, suggesting its prevalence may be underestimated [4]. BBSOAS presents with high clinical phenotypic heterogeneity. Ocular involvement is a common feature in affected individuals, manifesting primarily as optic atrophy, optic nerve hypoplasia, and brain-derived visual impairment. This distinct ocular phenotype differentiates BBSOAS from other NDDs [4, 13].

*NR2F1* is located on human chromosome 5q15 and encodes a 423 amino acid protein belonging to the orphan nuclear receptor family. NR2F1 has two highly conserved functional domains, a DBD and a ligand-binding domain (LBD), and two activation regions. While the ligand for the LBD remains unidentified, NR2F1 is known to bind to DNA structures via homo- or heterodimerization [14]. The DBD comprises two zinc finger motifs, ZF1 (aa86–106) and ZF2 (aa122–146), that interact directly with the DNA double helix [8]. NR2F1 is widely expressed in human tissues, including the brain, placenta, and ovaries. Impaired NR2F1 function can cause neurological disorders, possibly because it regulates neocortical morphology through region-specific modulation during neurodevelopment. Cortical morphology correlates strongly with brain malformations and NDDs [15–18]. Patients may exhibit visual dysfunction, suggesting that NR2F1 protein deficiency affects the visual system’s development. *Nr2f1* gene knockout mouse models show abnormal development of the optic papilla and late-onset axon guidance defects in neurons, characterized by reduced retinal ganglion cell density and disruption of axons projecting from the retina to the optic tract, leading to optic nerve hypoplasia [19].

The BBSOAS phenotype and genotype correlate. Bertacchi et al. found that variants within the NR2F1 DBD might cause more severe clinical manifestations, particularly language and motor function impairments [4]. The DBD has two highly conserved ZF structures, and variants within this region can significantly affect NR2F1 protein structure and function, disrupt dimer formation, and potentially cause dominant-negative effects [2]. The variant we identified, p.Met151Lys, is located within the DBD, and may create a new hydrogen bond with ZF1-Cys106, thereby reducing the stability of the ZF1 structure. Dual-luciferase assays showed that the p.Met151Lys variant significantly reduced the transcriptional activation activity of NR2F1, consistent with the functional impact trend reported by Chen et al. for the p.Ala155Thr variant near the DBD [3]. It is worth noting that the p.Ala155Thr variant is not located within the DBD but adjacent to its boundary, which may explain its relatively mild functional impact. Unlike the finding of Chen et al., p.Met151Lys did not appear to exhibit a dominant-negative effect (luciferase activity was not lower than that of the empty vector control). Therefore, although this variant may lead to severe clinical manifestations by disrupting the function of the DBD domain, its specific molecular mechanism requires further investigation. Additionally, variants in the start codon that restrict transcription and translation can cause severe phenotypes [20]. The LBD domain is crucial for NR2F1 protein dimer formation, but missense variants within this domain may manifest as milder clinical phenotypes [11].

BBSOAS has no specific treatment; management generally focuses on addressing symptoms. Tian et al. reported that a child with BBSOAS who received intramuscular injections of mouse nerve growth factor showed improved retinal electroretinography indicators and enhanced visual acuity [21]. However, the efficacy and safety of such interventions still need to be validated through large-scale studies. There are currently no effective treatment options for the neurodevelopmental phenotypes associated with BBSOAS. Antisense oligonucleotide-mediated gene therapy, which may regulate NR2F1 protein expression to partially restore function, has potential for therapeutic application, but its translational potential requires further assessment [4].

This study has several limitations. First, in vitro experiments are susceptible to experimental errors and may fail to mimic the in vivo or endogenous cell environments. Second, long-term follow-up data were lacking. Third, the severity of optic neuropathy was not analyzed. We recommend that the parents undergo high-resolution optical coherence tomography to monitor ocular disease progression.

## Conclusions

We diagnosed an infant with severe intellectual disability, motor impairment, and optic nerve pallor. Genetic testing identified a novel *NR2F1* gene variant that disrupts transcriptional activity, potentially affects the DBD-ZF structure, and causes severe neurological phenotypes. This study suggests that clinicians should be vigilant of possible BBSOAS in young children with NDD and associated optic neuropathy.

## Electronic supplementary material

Below is the link to the electronic supplementary material.

Supplementary Material 1

## Data Availability

No datasets were generated or analysed during the current study.
