# Supplementary material for: A pathogenic NR2F1 gene variant disrupts transcriptional activity and causes severe neurodevelopmental delay in Bosch-Boonstra-Schaaf syndrome
Source: Hereditas. 2025 Mar 1;162:30. doi: 10.1186/s41065-025-00394-8 (PMC11871647; doi:10.1186/s41065-025-00394-8)
Supplement: Supplementary file 1 — Supplementary Material 1 [file 41065_2025_394_MOESM1_ESM.docx]

**Supplementary Material 1.** Plasmid information constructed in this experiment.

|  | Primer sequence |
| --- | --- |
| NR2F1-WT-F | AAGGATGACGATGACAAGCTTATGGCAATGGTAGTTAGCAGCTG |
| NR2F1-WT-R | TGCTGGATATCTGCAGAATTCCTAGGAGCACTGGATGGACATGT |
| NR2F1-MUT-F | AAGGATGACGATGACAAGCTTATGGCAATGGTAGTTAGCAGCTG |
| NR2F1-MUT-R | TGCTGGATATCTGCAGAATTCCTAGGAGCACTGGATGGACATGT |
| NR2F1-MUT-T-R | CCTCtTGCCCACTTTGAGGCACTTCTTGAGGC |
| NR2F1-MUT-T-F | TCAAAGTGGGCAAGAGGCGGGAAGCGGTTCAG |


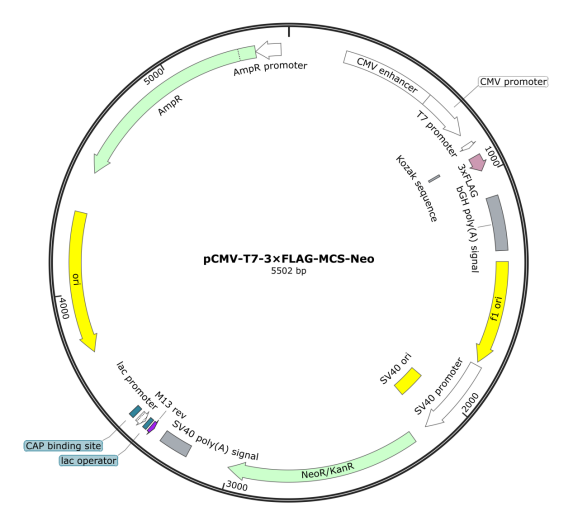

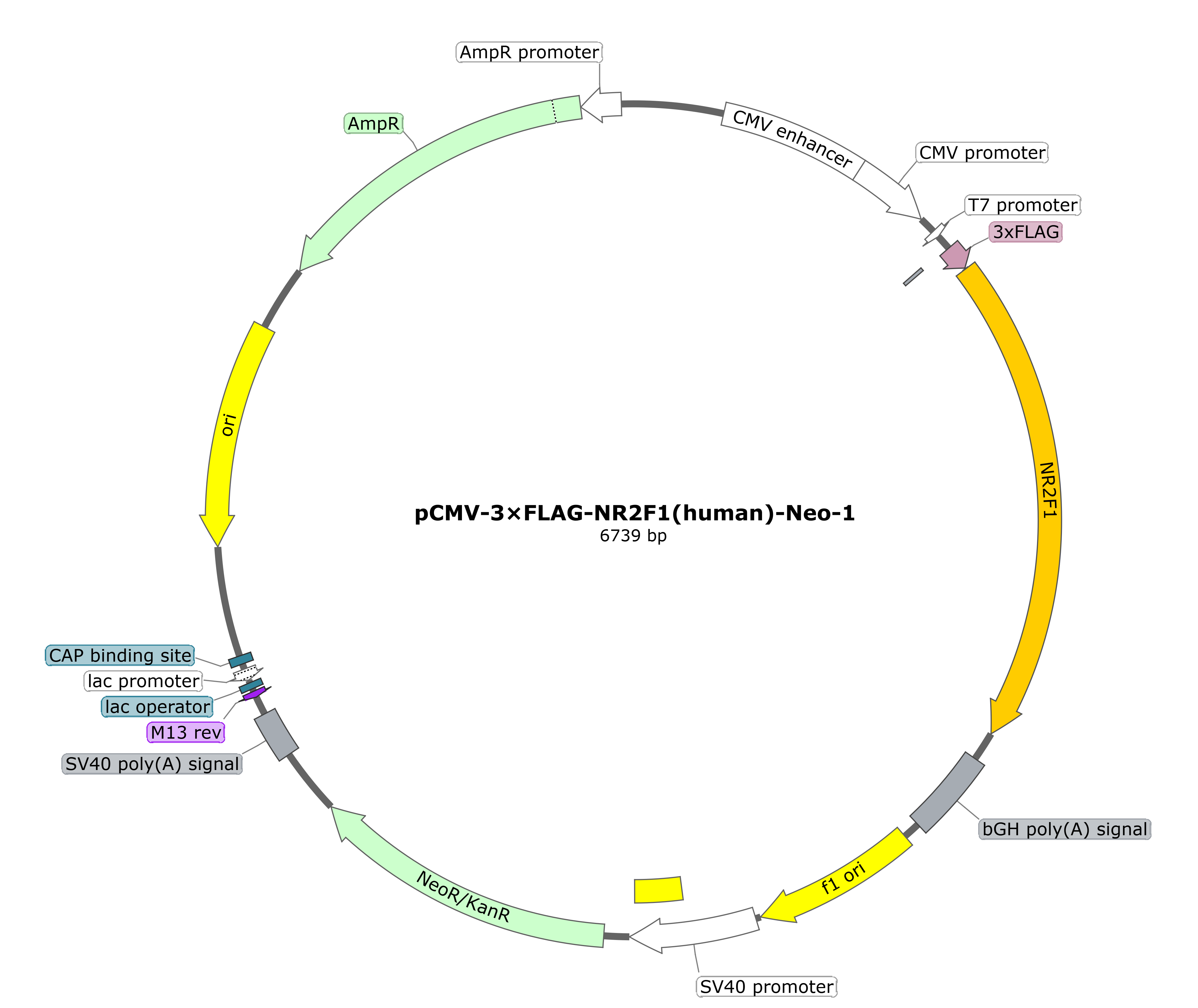


Figure. Cloning vector: pECMV-3×FLAG-N; Cloning sites: HindIII/EcoRI (On the left is an empty plasmid).


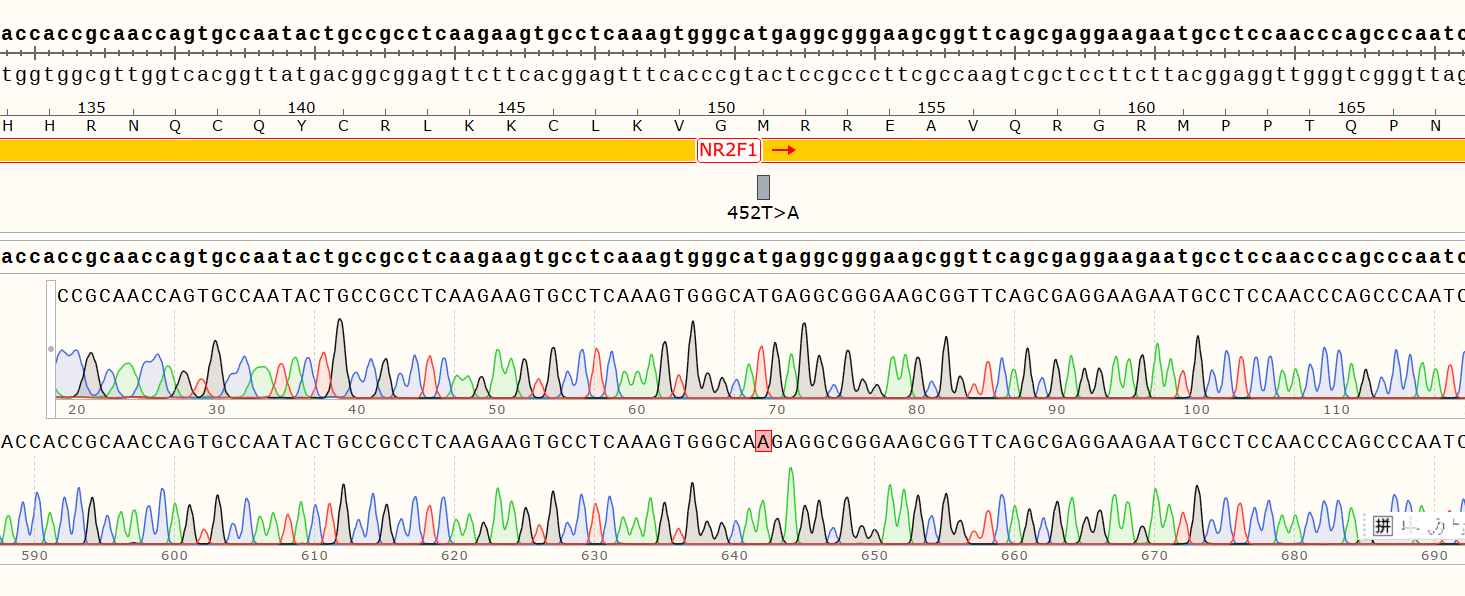


Figure. Result of the plasmid Sanger sequencing.

**Supplementary Material 2.** Sequencing data filtering strategy and related parameters in this study.
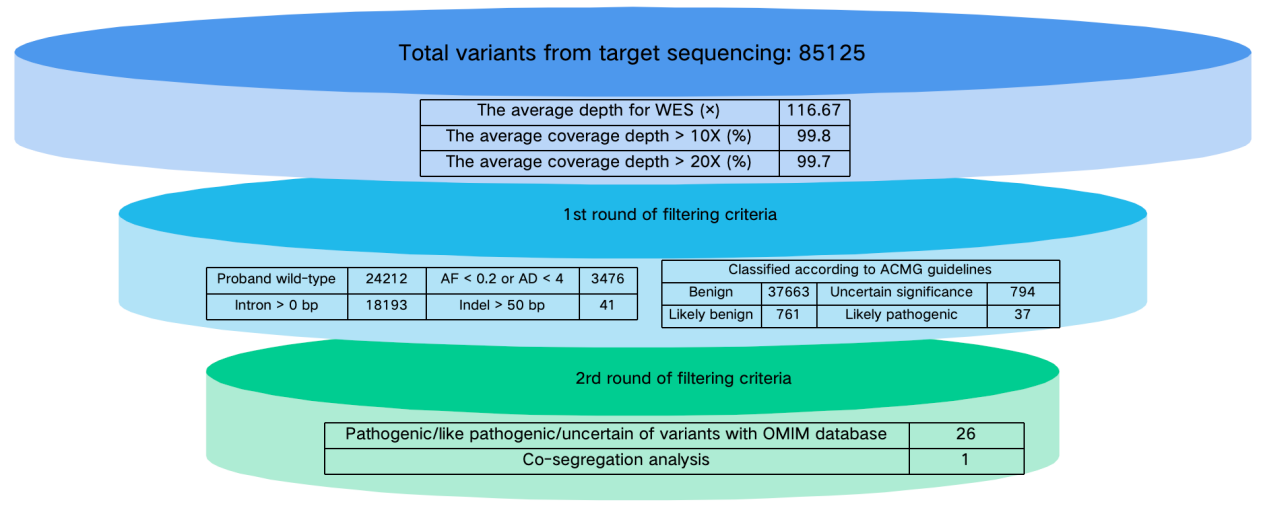
Figure. Funnel diagram of variation data filtering strategy.

**Supplementary Material 3**. *NR2F1* gene variants associated with BBSOAS as reported in the literatures.

| Patient | DNA variant (NM_005654.4) | Amino acid variant | Domain | Inheritance | Ref |
| --- | --- | --- | --- | --- | --- |
| 1 | c.339C>A | p.Ser113Arg | DBD | Germline | [1] |
| 2 | c.344G>C | p.Arg115Pro | DBD | Germline | [1] |
| 3 | c.755T>C | p.Leu252Pro | LBD | Germline | [1] |
| 4 | c.335G>A | p.Arg112Lys | DBD | Germline | [1] |
| 5 | c.2T>C | p.Met1? |  | Germline | [2] |
| 6 | c.403C>A | p.Arg135Ser | DBD | Germline | [2] |
| 7 | c.425G>T | p.Arg142Leu | DBD | Germline | [2] |
| 8 | c.2T>G | p.Met1? |  | Germline | [2] |
| 9 | c.328_330del | p.Phe110del | DBD | Germline | [2] |
| 10 | c.382T>C | p.Cys128Arg | DBD | Germline | [2] |
| 11 | c.2T>G | p.Met1? |  | Germline | [2] |
| 12 | c.463G>A | p.Ala155* |  | Germline | [2] |
| 13 | c.436T>C | p.Cys146Arg | DBD | Germline | [2] |
| 14 | c.2T>C | p.Met1? |  | Germline | [2] |
| 15 | c.413G>A | p.Cys138Tyr | DBD | Germline | [2] |
| 16 | c.1103G>A | p.Gly368Asp | LBD | Germline | [2] |
| 17 | c.291delC | p.His79Hisfs*22 |  | Germline | [2] |
| 18 | c.103_113delinCGCCGCCGC | p.Gly35Argfs*361 |  | Germline | [2] |
| 19 | c.2_4delinTGG | p.Met1? |  | Germline | [2] |
| 20 | c.1115T>C | p.Leu372Pro | LBD | Germline | [3] |
| 21 | c.257G>T | p.Cys86Phe | DBD | Germline | [3] |
| 22 | c.403C>T | p.Arg135Cys | DBD | Germline | [4] |
| 23 | c.286A>G | p.Lys96Glu | DBD | Germline | [5] |
| 24 | c.513C>G | p.Tyr171* |  | Germline | [6] |
| 25 | c.425G>A | p.Arg142His | DBD | Germline | [7] |
| 26 | c.2T>C; p.M1? | p.Met1? |  | Germline | [7] |
| 27 | c.729_730delinsCT | p.Gln244* | LBD | Germline | [7] |
| 28 | c.115G>T | p.Glu39* |  | Germline | [7] |
| 29 | c.292T>C | p.Tyr98His | DBD | Germline | [7] |
| 30 | c.967_968delAA; p.Ly | p.Lys323Serfs*73 | LBD | Germline | [7] |
| 31 | c.1080del | p.Asn362Thrfs*33 | LBD | Germline | [8] |
| 32 | c.82C>T | p.Gln28* |  | Germline | [9] |
| 33 | c.253G>T | p.Glu85* | DBD | Germline | [10] |
| 34 | c.313G>A | p.Gly105Ser | DBD | Germline | [11] |
| 35 | c.313G>A | p.Gly105Ser | DBD | Germline | [11] |
| 36 | c.256T>C | p.Cys86Arg | DBD | Germline | [12] |
| 37 | c.262 G>A | p.Val88Met | DBD | Germline | [12] |
| 38 | c.284G>T | p.Gly95Val | DBD | Germline | [12] |
| 39 | c.290A>C | p.His97Pro | DBD | Germline | [12] |
| 40 | c.293A>G | p.Tyr98Cys | DBD | Germline | [12] |
| 41 | c.311A>G | p.Glu104Gly | DBD | Germline | [12] |
| 42 | c.323G>T | p.Ser108Ile | DBD | Germline | [12] |
| 43 | c.365G>C | p.Cys122Ser | DBD | Germline | [12] |
| 44 | c.417A>T | p.Gln139His | DBD | Germline | [12] |
| 45 | c.1A>G | p.Met1? |  | Germline | [12] |
| 46 | c.2T>C | p.Met1? |  | Germline | [12] |
| 47 | c.380dupA | p.Asn127Lysfs*270 | DBD | Germline | [12] |
| 48 | c.1117C>T | p.Arg373* | LBD | Germline | [12] |
| 49 | c.931G>C | p.Ala311Pro | LBD | Germline | [12] |
| 50 | c.954G>C | p.Glu318Asp | LBD | Germline | [12] |
| 51 | c.1217T>C | p.Met406* |  | Germline | [12] |
| 52 | c.602C > A | p.Ser201* |  | Germline | [13] |
| 53 | c.2T>C | p.Met1? |  | Germline | [14] |
| 54 | c.4delG | p.Ala2Glnfs*3 |  | Germline | [15] |
| 55 | c.51_69dup | p.Asn24Glyfs*379 |  | Germline | [15] |
| 56 | c.91_93dupCGC | p.Arg31dup |  | Germline | [15] |
| 57 | c.115G>T | p.Glu39* |  | Germline | [15] |
| 58 | [c.290A>C](javascript:;) | p.His97Pro | DBD | Germline | [15] |
| 59 | [c.353T>G](javascript:;) | p.Leu118* | DBD | Germline | [15] |
| 60 | c.359dupA | p.Tyr120* | DBD | Germline | [15] |
| 61 | c.366C>G | p.Cys122Trp | DBD | Germline | [15] |
| 62 | [c.463G>A](javascript:;) | p.Ala155Thr |  | Germline | [15] |
| 63 | c.513G>C | p.Tyr171* |  | Germline | [15] |
| 64 | c.599C>G | p.Thr200Arg |  | Germline | [15] |
| 65 | c.698G>A | p.Trp233* | LBD | Germline | [15] |
| 66 | c.1024G>A | p.Glu342Lys | LBD | Germline | [15] |
| 67 | c.1036_1047del | p.Glu346_Gln349del | LBD | Germline | [15] |
| 68 | [c.1115T>C](javascript:;) | p.Leu372Pro | LBD | Germline | [15] |
| 69 | c.1118_1123del | p.Arg373_Leu374del | LBD | Germline | [15] |
| 70 | c.1183G>A | p.Gly395Ser |  | Germline | [15] |
| 71 | c.1198G>T | p.Glu400* |  | Germline | [15] |
| 72 | c.437G>A | p.Cys146Tyr | DBD | Germline | [16] |
| 73 | c.257-258delinsTT | p.Cys86Phe | DBD | Germline | [17] |
| 74 | c.403C > G | p.Arg135Gly | DBD | Germline | [17] |
| 75 | c.383G > A | p.Cys128Tyr | DBD | Germline | [17] |
| 76 | c.1065C>G | p.Tyr355* | LBD | mosaicism | [18] |
| 77 | c.169C>T | p.Gln57* |  | mosaicism | [19] |
| 78 | ~2.85Mb deletion | / |  | Germline | [1] |
| 79 | ~0.83Mb deletion | / |  | Germline | [1] |
| 80 | ~0.9Mb deletion | / |  | Germline | [2] |
| 81 | ~5.0Mb deletion | / |  | Germline | [2] |
| 82 | ~0.2Mb deletion | / |  | Germline | [2] |
| 83 | ~0.9Mb deletion | / |  | Germline | [2] |
| 84 | ~1.2Mb deletion | / |  | Germline | [2] |
| 85 | ~7.94Mb deletion | / |  | Germline | [3] |
| 86 | ~2.33Mb deletion | / |  | Germline | [12] |
| 87 | ~0.93Mb deletion | / |  | Germline | [12] |
| 88 | ~599kb deletion | / |  | Germline | [15] |
| 89 | ~0.582Mb deletion | / |  | Germline | [20] |

Abbreviations: DBD, DNA-binding domain; LBD, ligand-binding domain.

**References**

1. Bosch DG, Boonstra FN, Gonzaga-Jauregui C, et al. NR2F1 mutations cause optic atrophy with intellectual disability. *Am J Hum Genet*. 2014;94(2):303-9.
2. Chen CA, Bosch DG, Cho MT, et al. The expanding clinical phenotype of Bosch-Boonstra-Schaaf optic atrophy syndrome: 20 new cases and possible genotype-phenotype correlations. *Genet Med*. 2016;18(11):1143-1150.
3. Kaiwar C, Zimmermann MT, Ferber MJ, et al. Novel NR2F1 variants likely disrupt DNA binding: molecular modeling in two cases, review of published cases, genotype-phenotype correlation, and phenotypic expansion of the Bosch-Boonstra-Schaaf optic atrophy syndrome. *Cold Spring Harb Mol Case Stud*. 2017;3(6):a002162.
4. Hino-Fukuyo N, Kikuchi A, Yokoyama H, et al. Long-term outcome of a 26-year-old woman with West syndrome and an nuclear receptor subfamily 2 group F member 1 gene (NR2F1) mutation. *Seizure*. 2017;50:144-146.
5. Martín-Hernández E, Rodríguez-García ME, Chen CA, et al. Mitochondrial involvement in a Bosch-Boonstra-Schaaf optic atrophy syndrome patient with a novel de novo NR2F1 gene mutation. *J Hum Genet*. 2018;63(4):525-528.
6. Park SE, Lee JS, Lee ST, et al. Targeted panel sequencing identifies a novel NR2F1 mutations in a patient with Bosch-Boonstra-Schaaf optic atrophy syndrome. *Ophthalmic Genet*. 2019;40(4):359-361.
7. Bertacchi M, Romano AL, Loubat A, et al. NR2F1 regulates regional progenitor dynamics in the mouse neocortex and cortical gyrification in BBSOAS patients. *EMBO J*. 2020;39(13):e104163.
8. Walsh S, Gösswein SS, Rump A, et al. Novel dominant-negative NR2F1 frameshift mutation and a phenotypic expansion of the Bosch-Boonstra-Schaaf optic atrophy syndrome. *Eur J Med Genet*. 2020;63(10):104019.
9. Bojanek EK, Mosconi MW, Guter S, et al. Clinical and neurocognitive issues associated with Bosch-Boonstra-Schaaf optic atrophy syndrome: A case study. *Am J Med Genet A*. 2020;182(1):213-218.
10. Hobbs MM, Wolters WC, Rayapati AO. Bosch-Boonstra-Schaaf Optic Atrophy Syndrome Presenting as New-Onset Psychosis in a 32-Year-Old Man: A Case Report and Literature Review. *J Psychiatr Pract*. 2020;26(1):58-62.
11. Mio C, Fogolari F, Pezzoli L, et al. Missense NR2F1 variant in monozygotic twins affected with the Bosch-Boonstra-Schaaf optic atrophy syndrome. *Mol Genet Genomic Med*. 2020;8(7):e1278.
12. Rech ME, McCarthy JM, Chen CA, et al. Phenotypic expansion of Bosch-Boonstra-Schaaf optic atrophy syndrome and further evidence for genotype-phenotype correlations. *Am J Med Genet A*. 2020;182(6):1426-1437.
13. Zou W, Cheng L, Lu S, et al. A de novo nonsense mutation in the N-terminal of ligand-binding domain of NR2F1 gene provoked a milder phenotype of BBSOAS. *Ophthalmic Genet*. 2020;41(1):88-89.
14. Gazdagh G, Mawby R, Self JE, et al. A severe case of Bosch-Boonstra-Schaaf optic atrophy syndrome with a novel description of coloboma and septo-optic dysplasia, owing to a start codon variant in the NR2F1 gene. *Am J Med Genet A*. 2022;188(3):900-906.
15. Jurkute N, Bertacchi M, Arno G, et al. Pathogenic NR2F1 variants cause a developmental ocular phenotype recapitulated in a mutant mouse model. *Brain Commun*. 2021;3(3):fcab162.
16. Kocaaga A, Yimenicioglu S, Gürsoy HH. Novel NR2F1 variant identified by whole-exome sequencing in a patient with Bosch-Boonstra-Schaaf optic atrophy syndrome. *Indian J Ophthalmol*. 2022;70(7):2762-2764.
17. Liang Y, Wan L, Liu X, et al. Infantile epileptic spasm syndrome as a new NR2F1 gene phenotype. *Int J Dev Neurosci*. 2024;84(1):75-83.
18. van Renterghem V, Vilain C, Devriendt K, et al. Two siblings with Bosch-Boonstra-Schaaf optic atrophy syndrome due to parental gonadal mosaicism. *Eur J Med Genet*. 2023;66(4):104729.
19. Hrvatin N, Pereza N, Čaljkušić-Mance T, et al. Second Case of Gonadal Mosaicism and a Novel Nonsense NR2F1 Gene Variant as the Cause of Bosch-Boonstra-Schaaf Optic Atrophy Syndrome. *Clin Genet*. 2024;106(6):786-787.
20. Al-Kateb H, Shimony JS, Vineyard M, et al. NR2F1 haploinsufficiency is associated with optic atrophy, dysmorphism and global developmental delay. *Am J Med Genet A*. 2013;161A(2):377-81.
